# Dissipation

**Published:** 1904-01

**Authors:** G. S. Martin

**Affiliations:** Toronto Junction.


### DISSIPATION.

By G. S. Martin, D. D. S., Toronto Junction.

Read before Toronto Dental Society.

There are, taking the word in its wide sense, many forms of dissipation. To-night I refer especially to the dissipation or scattering (which is the literal meaning of the word) of energy, talents and time so prevalent in this age of hurry.

Take, for example, our reading. In these days of "the making of many books" the great danger is that our range of reading will be so promiscuous and diffused as to be worse than useless to us. Nothing is so diametrically opposed to true scholarship as the indiscriminate reading of current literature. Our fathers were men of fewer books, but had a larger percentage of good books than we. I recently heard ths form of mental dissipation compared with the dissipation of the morphine or cocaine fiend. There comes a time in the history of the person who forms this bad habit of constantly reading light literature when he will read nothing that will not produce in him a thrill of excitement, just as the victim of the drug habit must have his period of exaltation, no matter how intense the depression following. In both habits the after-effects are similar in some respects, loss of memory, brain power, and an ambition for better things generally. This habit of reading trash is easy to contract, and may take as great an effort of will-power to overcome as for many an inebriate to give up his cups.

In a profession making such phenomenal progress as is dentistry, you will agree with me htere is much to be read if we would keep ourselves from becoming back numbers. The dentist who neglects to read the dental journals and the best of the text-books on dental subjects will very soon find himself the companion of the men who never attend a convention—the rank outsider.

You will agree with me that the young graduate in any profession who hinks that his education is completed when he has had his sheepskin framed and his shingle painted and hung, is making a grievous mistake. Just that mistake is being made every year by young men who are graduated from our excellent School of Dentistry. Many a young dentist thinks he has completed the cycle of dental science, and for him there are no more worlds to conquer, except it be that he is anxious to acquire a practice in the shortest time possible. It has seemed to me that more could be done by our college professors towards impressing the students during their college course, and particularly during their final year, with the necessity for continued study after graduation, and with the desirability of every young man keeping himself in touch with his profession and up to date, by not only subscribing for two or three dental journals, but also by joining and attending his local and provincial dental societies.

The weeks and months during which a young dentist is waiting for his patronage to grow are, if he only knew it, invaluable to him in fitting himself for his life work. If during these idle hours habits of industry are formed along the lines of professional reading and scientific experimentation and research, the later years will not be as barren of usefulness to the profession as is the case with many of us. Our education is at fault if we are not taught to think things out for ourselves, instead of blindly accepting as gospel all that we find in journal or text-book or hear in college lecture-room. The professor who says, "Young men, think for yourseves," has given his students the best advice possible to be given to any one beginning study in a liberal profession. The young professional man esttling in a town or city is naturally extremely anxious to get acquainted, eagerly accepts every invitation to mingle with society as an opportunity to gain friends and incidentally patrons and patients. If he be a young man of good address and "sunny ways" he will soon findh imself wishing there were more nights in each week, that he might take in all that society offers him.

In addition to society, he may think it advisable to join two or three fraternal societies. In some of these, with his education and ambition, he will naturally become an officer, and much time will be necessary to keep in touch with the details of business dealt with. If he be religiously inclined, he will become prominent in his church and young people's society or Sunday-school, which is right in its place and in moderation. If he is ambitious to shine politically, the municipal campaign opens a tempting field to him, and he may aspire to the Council, or he may become School Trustee, or director on his local Public Library Board, all in his laudable ambition to be of service to his fellow townsmen. I have no word of condemnation for the desire to serve the public in these capacities, but simply to point out the dangers of dissipation, of "diffusiveness to the point of inefficiency," as some one has said.

In these days men are finding out that even one profession is too broad for them to possibly hope to excel in the practice of all its branches, and so the tendency of the age is towards specialization. In law we find the formation of large firms, composed of men each of whom devotes himself to one distinct branch of law, and thus becomes particularly efficient in its practice. In medicine we find the same conditions on the increase, and few physicians now pretend, except of necessity, to care for the whole human body. There is also a tendency, in dentistry, particularly in large centers of population, towards the development of specialties—prosthetic, orthodontia, extracting, pyorrhea treatment, implantation.

Surely if the most up-to-date men of our profession find it conducive to efficiency and profitable withal to confine themselves to the practice of one branch of dentistry, it is self-evident that one man cannot be a good all-round dentist and all that the times demand he should be in skill, etc., and at the same time be engaged in a half-dozen or more other lines, all diverse from dentistry and from each other.

It is a most important announcement that was made at the last meeting of this society, when the executive committee reported favorably on the offering of prizes for the encouragement of original lines of research in dental science. The sportsman with the shotgun may do havoc in the flock of sparrows, but it is the heavy rifle alone that will be effective with large game worth bringing out of the woods. If we want to produce anything worthy a place in the annals of our profession, we must specialize, study some particular line toward which our talent or fancy directs us, and it is only by such methods of work by such men as Black, Flagg, Ottolengui, Case Johnson, Peck, Price, Younger, and others of our contemporaries, not to mention many who have laid deep, broad foundations and then passed away, that dentistry has reached its present proud position among the liberal professions. It is only by a continuation of such self-sacrificing work that further progress will be made, and much that is dark and mysterious today will have the sunlight of science turned upon it. With the present stress of competition it is not fair that our best men should devote their time to such problems without some recompense for the loss. Any scientific research that will benefit humanity is, to my mind, a matter which it would, under proper conditions, be competent for the State to recognize and aid substiantally. Until that time shall come, we must undertake to share in the sacrifice by providing substantial prizes for such work as is worthy of a place, as original research. If I succeed in making any one present stop and think over how much he is contributing to the sum total of scientific dental knowledge, I shall feel repaid.

